# Recycled Aluminum Paraffin Composite for Passive Cooling Application in Buildings

**DOI:** 10.3390/ma18040728

**Published:** 2025-02-07

**Authors:** Gyorgy Thalmaier, Nicoleta Cobîrzan, Lucian V. Fechete-Tutunaru, Mugur Ciprian Balan

**Affiliations:** 1 Faculty of Materials and Environmental Engineering, Technical University of Cluj-Napoca, 103 Muncii blv., 400641 Cluj-Napoca, Romania; 2 Faculty of Civil Engineering, Technical University of Cluj-Napoca, 15 C. Daicoviciu, 400020 Cluj-Napoca, Romania; 3 Faculty of Automotive, Mechatronics and Mechanical Engineering, Technical University of Cluj-Napoca, 103 Muncii blv., 400641 Cluj-Napoca, Romania; lucian.fechete@auto.utcluj.ro (L.V.F.-T.); mugur.balan@termo.utcluj.ro (M.C.B.)

**Keywords:** paraffin composite, thermal response, environmental impact, clay brick

## Abstract

This paper presents a new composite phase change material made of paraffin and recycled aluminum sawing chips. Aluminum sawing chips were selected as a thermal conductivity enhancer, the volume fraction (~15%) of which, in the paraffin composite material, was minimized by selecting a fraction with shape that minimizes its packing density. Therefore, the particles remained in contact inside the composite, and thus, their sinking in the liquid state was avoided. The paraffin composite obtained had a latent heat capacity reduced by 20% compared to the paraffin and the thermal conductivity increased by 236%. The composite materials also had a wide active temperature range (33–65 °C). Incorporation of this paraffin composite in the brick cavities was tested as a way of improving the heat transfer fluctuations in the hot hours of the day. The experimental data show a maximum temperature difference of about 3 °C in comparison with bricks without the paraffin composite incorporated. To evaluate the environmental impact of the paraffin composite, the Global Warming Potential (CO_2_eq) was determined. The results highlighted less CO_2_eq in comparison with other recycled composite materials.

## 1. Introduction

The materials industry, in responding to climate neutrality and circular economy objectives [1,2,3], is facing new challenges in developing environmentally friendly, durable, and affordable materials at a low cost. Waste recycling represents an important pillar in achieving these objectives and requires the creation of functional synergy at the level of local and regional industries. The generated waste, if it meets the required quality standards, can stand as a valuable resource in the production of green or low-carbon materials. Most aluminum parts are delivered to the equipment manufacturers ready to use; no more machining is performed o n these parts. Aluminum producers generate waste up to 6% of the aluminum parts produced in the form of machining chips [4], which can be sold at only approx. 20% of the pure aluminum price [4]. Considering these facts, a different reuse strategy is proposed by the present paper, in which these chips can be used to manufacture phase change material (PCM) composites.

Phase change materials can potentially be used to increase the heat storage capacity of materials and buildings. During the phase change process, the heat is absorbed and stored [5,6] by materials, and then released inside the room with a delay in time, contributing to the thermal comfort and passive cooling of buildings [7].

PCM is thought to be efficient in reducing the energy demand for cooling lightweight buildings [5] or constructions located in hot environments. Efficiency depends on the season and the wall system [7,8] which is adopted.

Zhou et al. [6], in their review article, highlighted the types of PCM and their diversified applications in buildings. Some of the authors proposed and investigated experimental studies or numerical modeling of the applications of PCM to the mass of the materials [9,10]. Other authors proposed the immersion of PCM in the pores of aggregates of cladding materials [11], in the brick mass [12], or in their cavities [13].

Macro-encapsulated PCM was proposed for application, especially in walls facing south.

Castell et al. [5] carried out an experimental set-up test of several cubicles placed in Mediterranean climates. The cubicles’ walls were made of hollowed brick and blocks. Some of the cubicles were provided with PCM panels containing RT27-parafin or SP-25 A8 hydrate salt, which was macro-encapsulated and placed in the mass of the wall system. The experiments showed that the energy savings in the summer amounted to 17%.

Impregnation of porous materials with PCM was another measure studied by several researchers with the objective of increasing the stability of materials and walls, thus promoting and sustaining the passive cooling of indoor environments.

Kim et al. [9] proposed a new artificial stone for interior cladding. It was a composite material made of lightweight aggregates, carbon, and PCM with efficient heat storage capacity. Mahdaoui et al. [10], through a numerical analysis, determined the heat transfer of hollowed bricks with PCM imbued in the clay matrix. The results showed that PCM improved the storage ability of materials and significantly reduced the heat amplitude, which are important characteristics in energy efficiency.

Incorporation of PCM into the brick cavities was proposed as a simple way of to reduce the daily fluctuations in heat transfer. Vicente et al. [11], in their experimental studies, investigated wall specimens in which the brick cavity was filled with PCM with and without a thermal insulation layer. The results revealed that the thermal insulation layer in the wall system with PCM had a favorable effect in terms of decreasing the thermal amplitude.

Due to leakage of PCM liquid, the method of impregnation may be found to be inefficient in the case of porous materials exposed to different thermal conditions. In this regard, encapsulation may be a required and more efficient method. Khedache et al. [14] proposed and characterized the PCM composite materials produced by the dispersion method, in which contained paraffin, red brick, and expanded graphite in their compositions in different percentages. The thermal conductivity was increased due to the presence of graphite in an amount up to three times that of paraffin, consequently decreasing the latent heat storage capacity.

Due to the low thermal conductivity, most of the PCMs have long charging–discharging periods [6], which can be improved by adding different additives (aluminum, metal foam, expanded graphite, carbon fiber). This process is effective in increasing the speed at which they store and release heat, but may have consequences such as increases in weight and system cost. A weight increase represents a drawback, especially in the case of multistorey buildings placed in seismic areas.

Recently, two ways have been proposed to increase the thermal conductivity of the PCMs, one by using a metallic foam skeleton or fins and the second by incorporating nanoparticles. Nanoparticles are needed since bigger particles will sink during the melting of PCM. Both ways increase the costs and can affect negatively health and the environment.

The paper presents a simple and environmentally friendly way of increasing the paraffin’s thermal conductivity and consequently improving the charging–discharging characteristics of the resulting PCM composites by incorporating recycled aluminum sawing chips material from sawing aluminum bars. The novelty of the proposed solution lies in utilizing particles that have very low apparent density, which will greatly reduce the sinking tendency of the particles during the melting stage. This composite material has been proposed for infilling the cavities of some commercially available clay-fired bricks to study their potential to be used for passive cooling design.

## 2. Materials and Methods

**Figure 1:**
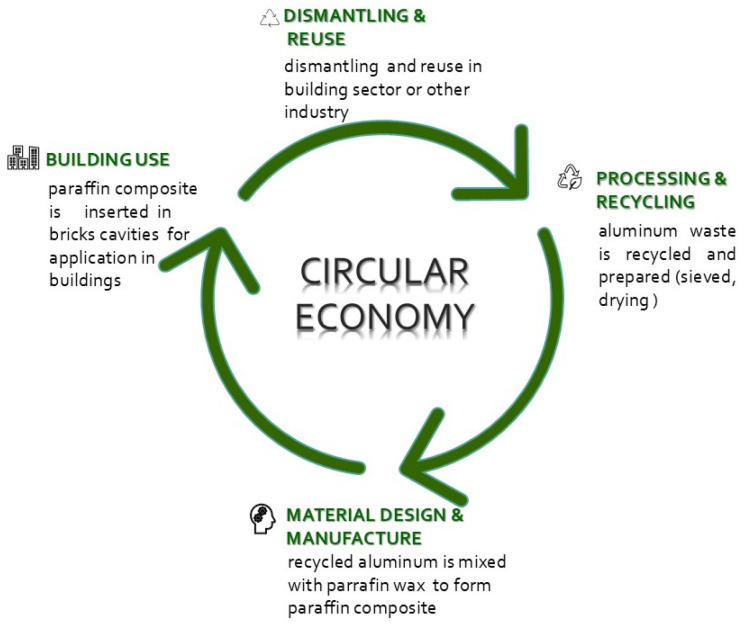
Schematics boundaries of the experimental setup.

The boundary of experimental setup, settled at the laboratory scale, is presented in Figure 1. It is based on circular principles and consists of recycling and processing aluminum waste, design and manufacture of composite materials, and insertion into bricks cavities. Bricks with PCM-infilled cavities are investigated to highlight their potential to be used as a sustainable solution passive in the cooling design of buildings.

A composite PCM based on paraffin with a melting temperature of 56 °C and recycled aluminum alloy (5754 series) sawing chips ~15% vol. was developed. Recycled aluminum was selected to increase the thermal conductivity of PCM and to achieve a higher volume percentage to limit the sinking in the liquid PCM due to mechanical contact between the metallic particles. To maximize the paraffin fraction, the chips were separated according to their shapes using a series of sieves (four sieves with mesh openings of 125 µm), and I knew that the residence time of each particle on a sieve was strongly dependent on its shape [15]. For each fraction, the apparent density was determined, and the particles with the lowest apparent density were used in the successive experiments. The apparent density from the selected fraction was 0.41 g/cm^3^, in order to maximize the paraffin content and, consequently, the heat absorption. The appropriate quantities of components were heated to 70 °C in a water bath of a constant temperature, maintained for 5 min to ensure a uniform temperature and a low viscosity, and then mixed until almost solidified to manufacture the composite according to the melt blending method. There was no need for ultrasonication or other more complex homogenization due to the size of the spaces between the particles and their good wetting by the paraffin.

**Figure 2:**
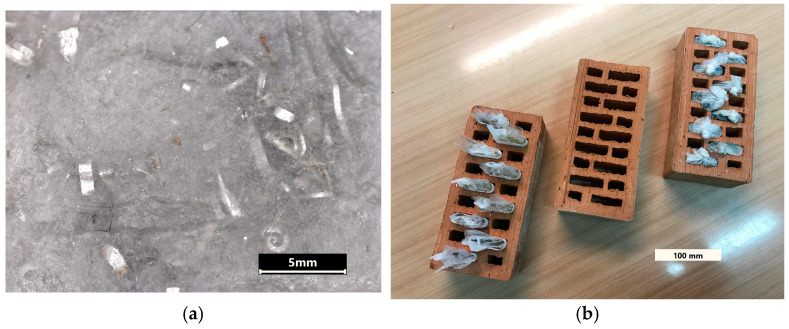
(**a**) Optical microscopy image of the manufactured paraffin composite, and (**b**) the tested brics with and without the composite.

The resulting PCM composites (Figure 2a) were infilled into one-brick cavities (PCM-filled specimens Figure 2b), and their performance was evaluated and compared with control bricks. The two bricks were insulated from each other and from the outside environment with a layer of expanded polystyrene that was 100 mm thick. The assembly was heated on the front surface with a heat flux of 0.6 W/m^2^, and the temperature variation was monitored by non-contact IR thermal imaging. Images were taken every 15 min on the front and back sides of the bricks. The obtained images were analyzed, and the temperature variation with time was evidenced.

Bricks are porous material, and the major source of concern in PCM-filled bricks is their tendency to leak the melted PCM. A simple way to protect against this type of defect is the encapsulation of the phase change material. In that regard, a thin bag of polyethylene can be used as an encapsulating material due to its low cost and availability.

The melting and freezing behavior of the paraffin and the manufactured paraffin composite were evaluated using differential scanning calorimetry in the air with a heating rate of 2 K/min.

Specific heats (Cp) were estimated using the rule of mixtures. The thermal diffusivity, a thermal parameter which describes the rate of heat transfer in a material from the hot to the cold side, was then calculated by dividing the thermal conductivity, the density, and the specific heat capacity [16] of the materials.

The thermal conductivity values of the composites were measured by a stationary temperature field method on a thermal conductivity experimental setup, which is detailed elsewhere [17].

Prior to the measurements, the steady-state unidirectional heat flow was achieved and maintained for at least 30 min. To minimize the heat loss during the measurements, the whole experimental set-up was isolated from the environment with 10 cm basalt wool with a thermal conductivity better than 0.033 W/mK. The experimental uncertainty was around 5–7%. Every measurement was repeated three times, and the presented value was their mean value.

The resulting PCM composites were infilled in one brick cavity (PCM-filled specimens) and their performance was evaluated and compared with the control bricks. The two bricks were insulated from each other from the outside environment, with a layer of 100 mm thick expanded polystyrene. Bricks are porous material, and the major source of concern in PCM-filled bricks is their tendency to leak the melted PCM. A simple way to protect against this type of defect is the encapsulation of the phase change material. In that regard, a thin bag of polyethylene was used as an encapsulating material due to low cost and availability.

The assembly was heated on the front surface, with a commercially available electric heat source with variable output. The heat flux was monitored, set to 0.6 W/m^2^, and the temperature variation was monitored by non-contact IR thermal imaging. Images were taken every 15 min on the front and back sides of the bricks for 6 h. The obtained images were analyzed, and the temperature variation with time was evidenced.

Although the environmental impact of the production processes were highly plant-dependent, and only on-site emissions and the emissions generated by the production of the required electric energy were considered. These two are dominant in the whole production chain. The values given by literature [18,19] were compared with the measured energy used to clean, mix, and melt the samples. These values were then converted to equivalent CO_2_ emissions using the conversion factor presented by the UK government for the year 2022 [20].

## 3. Results and Discussion

### 3.1. Thermal Characterisation of PCM Composite

To maximize the PCM fraction in the composite, one must ensure that the percentage of aluminum particles is minimized. The limiting factor is their tendency for sedimentation in the liquid phase. To have an extremely low sedimentation speed that makes them usable in the long term, the particle size should be in the nanometer range, according to Stokes’ law for sedimentation. The manufacturing of aluminum (and other) particles of this size is possible; however, the costs and the other environmental problems are making these nanoparticles unsuitable for this kind of application. Another simpler solution to the sedimentation problem is to use low-packing-density particles. The particles’ lowest density is their apparent density. This density is dependent on the interparticle friction and on the particle shape. The more the particle shape differs from a sphere (the shape that gives usually the highest apparent density), the lower the apparent density is.

**Figure 3:**
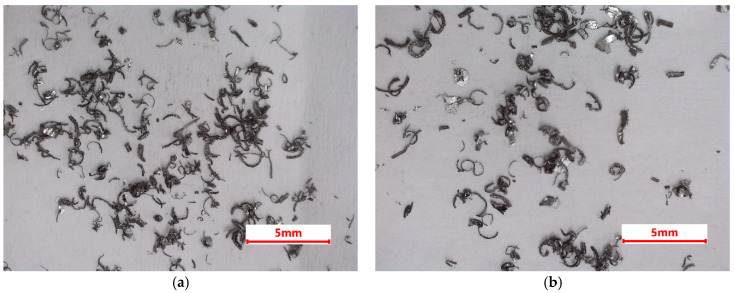

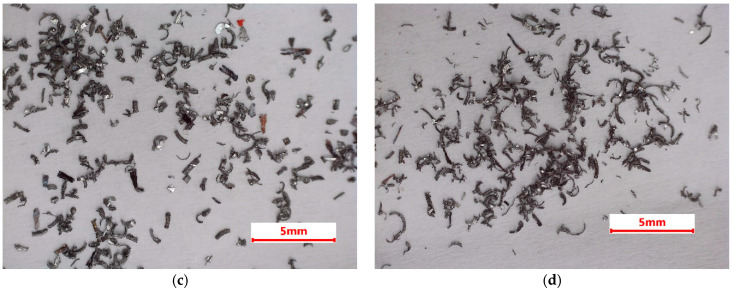
Stereoscopic optical images of the separated particles: (**a**) sieve 1, (**b**) sieve 2, (**c**) sieve 3, (**d**) sieve 4.

The chips have particles of different shapes, from round particles to curved, needle-type ones. To minimize the apparent density, we separated the latter types of particles by sieving. It is known that, by using a sieve cascadograph (a series of sieves with the same mesh size) [13], a good separation of the particle shape can be achieved, since particles that have rounded shapes can pass significantly faster through the sieves than particles with high shape anisotropy (Figure 3).

Their apparent density varied from 0.41 g/cm^3^ for sieve 1, with the particles that passed the slowest through the mesh openings, the particles on the following sieve which had high shape factors, too; the particles that bent less than the previous ones had a density of 0.42 g/cm^3^, the particles from the third sieves had the lowest elongation and the highest apparent density of all (0.51 g/cm^3^); and the last batch of particles that passed through all the sieves was thinner and usually smaller. The apparent density was 0.46 g/cm^3^. As a comparison, one can look at the powder’s specifications of different powder producers that make powders for the powder metallurgy. For example, Hoganas was offered atomized aluminum powders for 3D printing with a typical apparent density of 1.3 g/cm^3^ [21].

**Figure 4:**
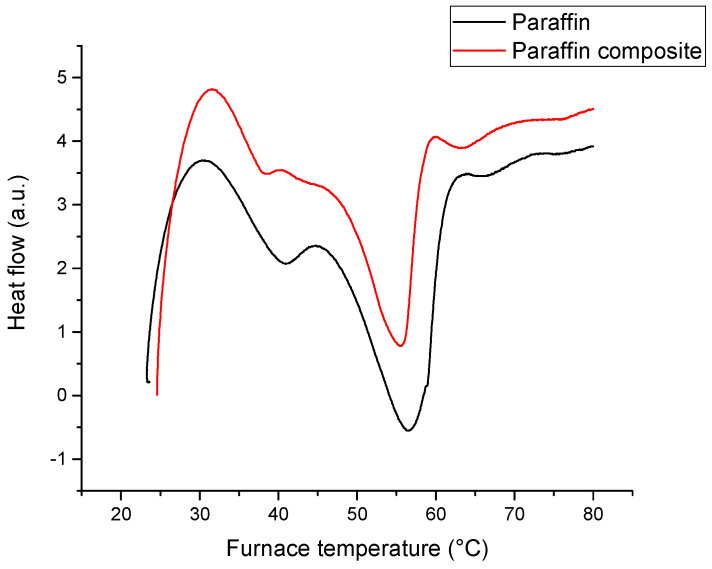
DSC heating curves (heating rate of 2 °C/min).

The phase change behavior of composite materials was studied by differential scanning calorimetry; the onset and peak temperatures were used to describe the melting and solidification behavior. Well-defined peaks may be seen in Figure 4, corresponding to melting and solidification events. Since only the paraffin underwent thermal transformations, the peaks corresponding to the PCM composites have smaller areas per unit mass compared to the pure PCM. As one can see from the data in Table 1, the presence of the metallic particles shifted the peak position in the DSC curves.

**Figure 5:**
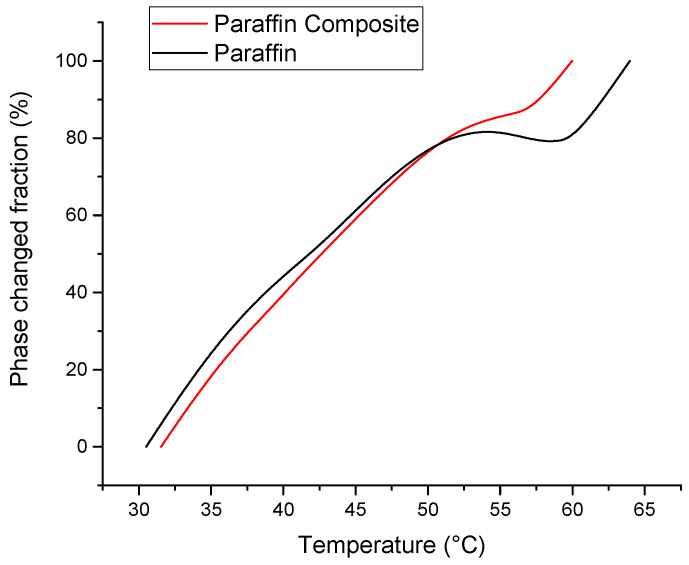
Phase change fraction as a function of temperature at a heating rate of 2 °C/min.

**Figure 6:**
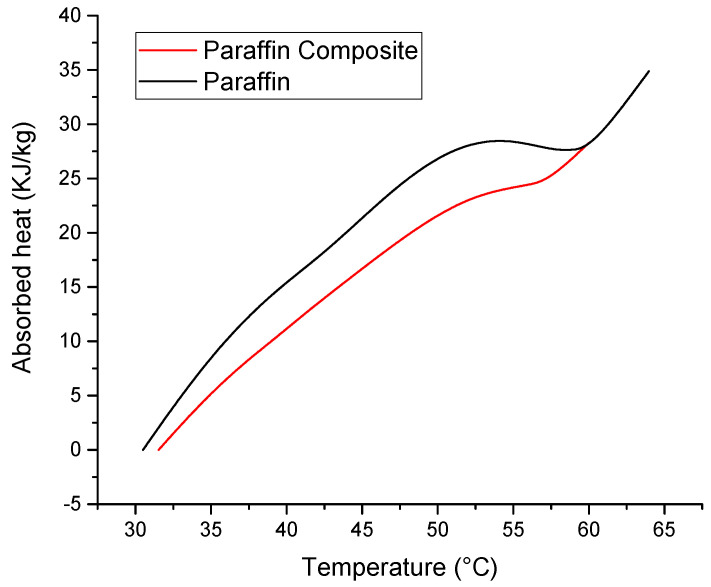
Cumulative heats as function of temperature determined by numerical integration of the melting DSC peak areas.

A simple way of evidencing the latent heat exchange is based on the DSC peaks. By integrating, it can be easily calculated as the fraction of the phase change with temperature and the absorbed or released heats (at 35 °C 16% of the total energy can be absorbed, at 45 °C 52% and at 55 °C 85% of the total absorbable energy is absorbed. The paraffin composite is completely melted at 60 °C). These results are presented in Figure 5 and Figure 6.

Paraffin has low thermal conductivity, and so its reaction to temperature modifications in the environment is also slow. By incorporating the metallic aluminum chips into the paraffin matrix, a significant increase in the thermal conductivity of the composites was observed. The loose aluminum chips had high porosity due to the particle shape, which did not facilitate good particle packing.

The thermal parameters calculated with the methodologies presented in Section 2 may be seen in Table 1.

The thermal conductivity, in the case of the un-sintered aluminum chips, increases 4 times, although there is no good thermal contact between the particles. The increase in thermal conductivity is at the expense of the latent heat capacity which is reduced to close to 80% of that of the pure paraffin, since the metallic particles are not taking part in these phase changes. The specific heat is also dependent on the metal content, a decrease being caused by the added metal fraction, which has a lower specific heat value. Other metals were analyzed by different researchers due to their ability to conduct heat and activate the PCM composites. This enhances the thermal conductivity of PCMs being dependent on the type and fraction ratio of admixture (Table 2).

Thermal diffusivity of other thermal parameters of the composite is improved with heat passing through the composite, making the melting and freezing behavior faster.

The thermal diffusivity coefficient is almost double in cases of PCM composites, meaning that the PCM capacity is charging you if you; if exchange the thermal energy with its environments, it has been improved.

### 3.2. Thermal Characterisation of Bricks with PCM

The efficiency of integration of phase change materials (PCMs) into bricks as a promising method in passive cooling design of buildings was analyzed.

**Figure 7:**
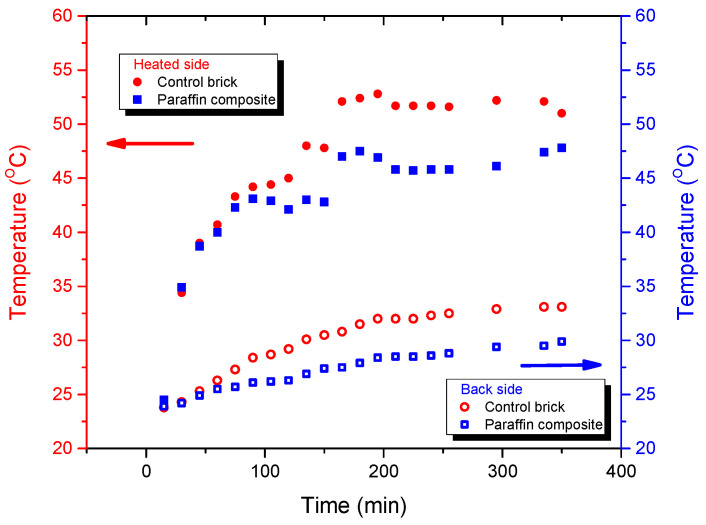
Temperature increases in the bricks on heating with and without PCM.

The performance of bricks with PCM composites was measured in a laboratory and compared with a control brick without PCM (Figure 7). The results are presented in the figure listed below.

Slower temperature increases for the brick containing the PCM composite were observed on both face sides (heated and unheated). The heating rate was lowered initially by the PCM’s higher heat capacity, and later by its phase change. The wide melting range of the PCM composite permits the material to work efficiently in a wide temperature interval. A maximum temperature difference of 3 °C was measured after 6 h of heating.

Based on thermal characteristics of composite PCMs, as presented in Table 1, the measures obtained led to the determination of these strong thermal parameters of bricks (control and bricks with PCM)—Table 3.

The results show that the thermal conductivity of a standard brick is 0.24 W/mK, while the brick with PCM reaches the value of 0.47 W/mK in a solid state and decreases up to 0.238 due to heat absorption, which is in good agreement with the DSC curve.

Thermal inertia of building elements is also an important parameter in passive cooling design. A reduction in the energy consumption in buildings may be obtained due to increasing thermal inertia and control of the daily temperature fluctuations.

### 3.3. Environmental Impact

Aluminum is a technical metal of utmost importance and is used in every branch of industry. Its life cycle starts with the mining of the ore. Then, the ore is transformed into a primary ingot by the Bayer process, followed by electrolysis. Secondary aluminum is obtained by smelting scrap aluminum alloys. The life cycle continues with the semi-manufacturing and manufacturing of a usable product. They are used, and after their useful life cycles, they undergo the final recycle or waste management [26]. It is estimated that a good portion of all aluminum extracted up to now is still in productive use today [27], so recycling of aluminum is conducted extensively. As we delve further into the production stage, it becomes more difficult to separate the scrap, so the collecting step of the recycling is becoming less reliant. For each method of producing aluminum (primary or secondary), huge energy input is required. By far the most energy intensive method is primary aluminum production, with more than double the energy required compared to the aluminum obtained from scrap [28].

Compared with melting, solid-state recycling of aluminum chips is considerably less energy-intensive, with up to four times less energy required [29] and thus is a preferable way, since the required mechanical properties are at a reasonable level.

**Figure 8:**
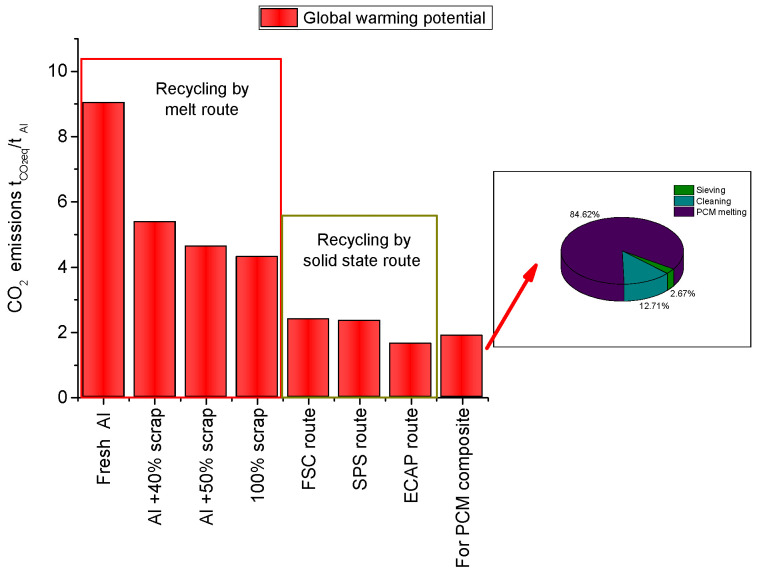
Reduced environmental impact due to almost no processing requirements.

The Global Warming Impact generated in these processes is highly plant-dependent. The comparison [21] presented in Figure 8 considers mean values given by the literature and only regarding the direct, on-site emissions, as well as emissions generated by the production of the required electric energy. These two were dominant in the whole production chain. Here, the same observations are valid as in the case of the energy input where the solid-state processing of the scrap was preferred to the melting-based processing.

Compared with this, the simple reuse of the aluminum scrap with minimal or without any subsequent processing was more energy-saving and subsequently saved them more environmental friendliness.

## 4. Conclusions

Increasing material circularity in the context of European Green Deal objective… this paper analyzed the feasibility of using aluminum sawing chips as secondary raw materials to produce a new paraffin-based composite material.

This work demonstrates that it is possible to separate particles with shapes that assure a low packing density in an extremely simple way by sieving. The sawing chips permit the separation of particles with an extremely low packing density of 0.41 g/cm^3^, almost 3 times lower than in the case of ordinary powders used in powder metallurgy.

In this study, 15% vol. waste aluminum was used as the thermal conductivity enhancer in a paraffin-based phase change composite. The obtained composite had good thermal conductivity, and was comparable with results obtained by using nanoparticles that had higher cost and may pose health concerns.

This composite was inserted into the bricks cavities to improve the thermal properties of materials and to improve efficiency in passive cooling application. The results showed that aluminum sawing chips were efficient in increasing the thermal conductivity of paraffin composite by 236% and reducing the internal temperature by about 3 °C when incorporated into the bricks cavity. Using these aluminum sawing chips is a good way to improve the thermal response of the paraffin-based PCM composites. When incorporating the aluminum chips into the paraffin matrix, a significant increase in the thermal conductivity of the composites was observed, and a reduction in latent heat capacity.

The thermal diffusivity of the PCM composite material was improved with up to 60% as the heat was transmitted through the composite, improving the melting and freezing behavior.

The waste material did not require special treatment and only marginally increased the embodied energy of the new PCM composite. These materials could be used in the construction industry due to their low cost, reasonable density, reduced health hazards, and limited environmental impact.

As the next step, real-life testing should be in order to ensure the long-term stability of the composite and the measurement of the energy saving for the cooling needs of a houses placed in different climatic conditions.

## Figures and Tables

**Table 1:** Thermal properties of the paraffin and its composite.

Material	Melting		Solidification		Variation of LHC %	Specific Heat (J/kgK)	Thermal Conductivity (W/mK)	Thermal Effusivity (Ws^1/2^ m^−2^ K^−1^)	Thermal Diffusivity (m^2^/s)	Density (kg/m^3^)
	T Onset °C	T Peak °C	T Onset °C	T Peak °C						
Paraffin	47.4	56.7	51.8	45.6	100	2144	0.25	682	1.34 × 10^−7^	869
PCM composite	47.7	55.7	51.8	47.5	80	1925	0.84	1361	3.81 × 10^−7^	1145

**Table 2:** Thermal conductivities of some paraffin composites.

Material	Fraction (%)	Thermal Conductivity Increase (%)	Reference
PCM composite	15% vol.	236% (0.25 to 0.84 W/mK)	this study
Paraffin silica-graphene oxide composite	15% mass	332% (0.2 to 0.84 W/mK)	[22]
Paraffin nanosilica-graphene nanopellets composite	1 + 1% mass	100% (0.24 to 0.48 W/mK)	[23]
copper foam/paraffin composite	90PPI	1170% (0.2 to 2.54 W/mK)	[24]
nano graphene/paraffin composite	3% mass	146% (0.123 to 0.303 W/mk)	[25]

**Table 3:** Thermal properties of the brick with and without PCM materials.

Material	Specific Heat (J/kgK)	Thermal Conductivity (W/mK)	Thermal Effusivity (Ws^1/2^ m^−2^ K^−1^)	Thermal Diffusivity (m^2^/s)	Thermal Inertia	Density (kg/m^3^)	Mass (kg)
Hollow brick	840	0.24	464	2.67 × 10^−7^	4.11	1070	1.9
Brick + 13.5 mass % paraffin (solid)	1040	0.30	618	2.35 × 10^−7^	4.38	1223	2.1
Brick + 13.5% mass PCM Composite (measured)	991	0.238	546	1.89 × 10^−7^	4.88	1265	2.2

## Data Availability

The original contributions presented in this study are included in the article. Further inquiries can be directed to the corresponding author.
